# Pancreatic Serous Cystic Neoplasms and Mucinous Cystic Neoplasms: Differential Diagnosis by Combining Imaging Features and Enhanced CT Texture Analysis

**DOI:** 10.3389/fonc.2021.745001

**Published:** 2021-12-23

**Authors:** Hai-Yan Chen, Xue-Ying Deng, Yao Pan, Jie-Yu Chen, Yun-Ying Liu, Wu-Jie Chen, Hong Yang, Yao Zheng, Yong-Bo Yang, Cheng Liu, Guo-Liang Shao, Ri-Sheng Yu

**Affiliations:** ^1^ Department of Radiology, Cancer Hospital of the University of Chinese Academy of Sciences (Zhejiang Cancer Hospital), Hangzhou, China; ^2^ Institue of Cancer and Basic Medicine (ICBM), Chinese Academy of Sciences, Hangzhou, China; ^3^ Department of Radiology, Second Affiliated Hospital, Zhejiang University School of Medicine, Hangzhou, China; ^4^ Department of Pathology, Cancer Hospital of the University of Chinese Academy of Sciences (Zhejiang Cancer Hospital), Hangzhou, China; ^5^ Research Institute of Artificial Intelligence in Healthcare, Hangzhou YITU Healthcare Technology Co. Ltd., Hangzhou, China; ^6^ Clinical Research Center of Hepatobiliary and Pancreatic Diseases of Zhejiang Province, Hangzhou, China

**Keywords:** pancreatic neoplasms, serous cystadenoma, mucinous cystadenoma, texture analysis, tomography

## Abstract

**Objective:**

To establish a diagnostic model by combining imaging features with enhanced CT texture analysis to differentiate pancreatic serous cystadenomas (SCNs) from pancreatic mucinous cystadenomas (MCNs).

**Materials and Methods:**

Fifty-seven and 43 patients with pathology-confirmed SCNs and MCNs, respectively, from one center were analyzed and divided into a training cohort (n = 72) and an internal validation cohort (n = 28). An external validation cohort (n = 28) from another center was allocated. Demographic and radiological information were collected. The least absolute shrinkage and selection operator (LASSO) and recursive feature elimination linear support vector machine (RFE_LinearSVC) were implemented to select significant features. Multivariable logistic regression algorithms were conducted for model construction. Receiver operating characteristic (ROC) curves for the models were evaluated, and their prediction efficiency was quantified by the area under the curve (AUC), 95% confidence interval (95% CI), sensitivity and specificity.

**Results:**

Following multivariable logistic regression analysis, the AUC was 0.932 and 0.887, the sensitivity was 87.5% and 90%, and the specificity was 82.4% and 84.6% with the training and validation cohorts, respectively, for the model combining radiological features and CT texture features. For the model based on radiological features alone, the AUC was 0.84 and 0.91, the sensitivity was 75% and 66.7%, and the specificity was 82.4% and 77% with the training and validation cohorts, respectively.

**Conclusion:**

This study showed that a logistic model combining radiological features and CT texture features is more effective in distinguishing SCNs from MCNs of the pancreas than a model based on radiological features alone.

## Introduction

Pancreatic serous cystic neoplasms (SCNs) originate from cuboidal epithelial cells full of glycogen-rich components, and are the only benign tumors of the pancreas, accounting for 10-16% of pancreatic cystic neoplasms (1, 2). The detection of SCNs is increasing, owing to the more widespread use of abdominal imaging (3); typically, however, SCNs only constitutes approximately 30% of all SCNs, presenting with a microcystic appearance with a star-like fibrous central scar with or without calcifications (4). Furthermore, as there is a chanceful spectrum of performances for SCNs in radiology, up to 60% of SCN patients performed surgery with uncertain diagnosis (5); atypical SCNs may misdiagnosed as mucinous cystic neoplasms (MCNs) or intraductal papillary mucinous neoplasms (IPMNs), which have the potential for malignancy, so misdiagnosis can lead to unnecessary surgery (6–8).

There is no consensus regarding the management of SCNs in terms of follow-up and surgery (9, 10). Symptoms, initial tumor size and growth rate are always taken into consideration when determining whether surgery should be performed (11–13). Some studies recommend surgery for SCNs measuring >4 cm regardless of the symptoms due to its rapid growth rate and high risk of symptoms onset (12), while others suggest resection only for SCNs with associated symptoms (10). SCNs are very safe and develop an indolent nature after long-term follow-up, while MCNs should be treated with surgery once a diagnosis is made (14).

Imaging examinations such as CT and MRI, are a mainstay in distinguishing SCNs and MCNs; however, their performance remains unsatisfactory. The accuracy in discriminating certain types of pancreatic cystic neoplasms is between 40-95% for MRI and between 10-81% for CT (10). Texture analysis is a popular technique for quantitatively assessing the heterogeneity of tissues through the extraction, analysis, and interpretation of radiological features and has been widely used in the treatment of pancreatic lesions, such as differential diagnosis, tumor grading, and prognosis prediction (15–18). Several studies related to the discrimination of SCNs and MCNs or that differentiate SCNs from other pancreatic cystic lesions (PCLs) using radiomics have been published (15, 19–24). However, these studies are limited by the use of single center data and small sample sizes, so multicenter studies with larger sample are urgently needed to clarify the role of radiomics. Hence, our study included the largest sample size to date and is the first to incorporate outside data to validate the performance of selected features. Then, we combined imaging features and enhanced CT texture analysis to distinguish SCNs from MCNs.

## Materials and Methods

### Patient Population

Ethical approval for this study was approved by the Second Affiliated Hospital of Zhejiang University School of Medicine and Zhejiang Cancer Hospital, and the requirement for informed consent was waived. We retrospectively collected patients diagnosed with pathologically confirmed SCNs (n = 57) or MCNs (n = 43) from the Second Affiliated Hospital of Zhejiang University School of Medicine from January 1, 2010 to October 30, 2019. A cohort to be used solely for external validation was collected from Zhejiang Cancer Hospital from January 1, 2009 to February 20, 2021 (SCN = 19, MCN = 9).

The inclusion criteria were as follows: (1) abdominal contrast-enhanced CT scan performed within 2 months before the operation; and (2) lesion diagnosis confirmed by surgery or biopsy. The exclusion criteria were as follows: (1) incomplete imaging or clinical information; (2) a lesion too small (≤ 5 mm) to draw a region of interest (ROI); and (3) poor image quality or contamination of the ROI by artifacts, preventing analysis.

### Image Acquisition

All patients fasted from solid food for approximately 4-6 hours before the examinations. The CT scans were performed with the following equipment: Siemens Somatom definition AS 64、Perspective (Siemens Medical Systems), TOSHIBA Aquilion 320 (TOSHIBA Medical Systems Corporation), and Optima CT680 Series (GE Medical Systems). The imaging parameters were as follows: kVp/effective mA = 120 Kv/160-250 mAs, slice thickness = 5 mm; and field of view = 320-380 mm. A plain scan was performed, followed by intravenous injection of nonionic contrast medium (Omnipaque 300 g/l; GE Healthcare; iopromide; Ultravist 370, Bayer Schering Pharma, 120 mL) at a rate of 3 mL/s. Images were obtained in the arterial phase (23-25 s), portal venous phase (40 s) and equilibrium phase (70 s).

### Image Analysis

**Figure 1:**
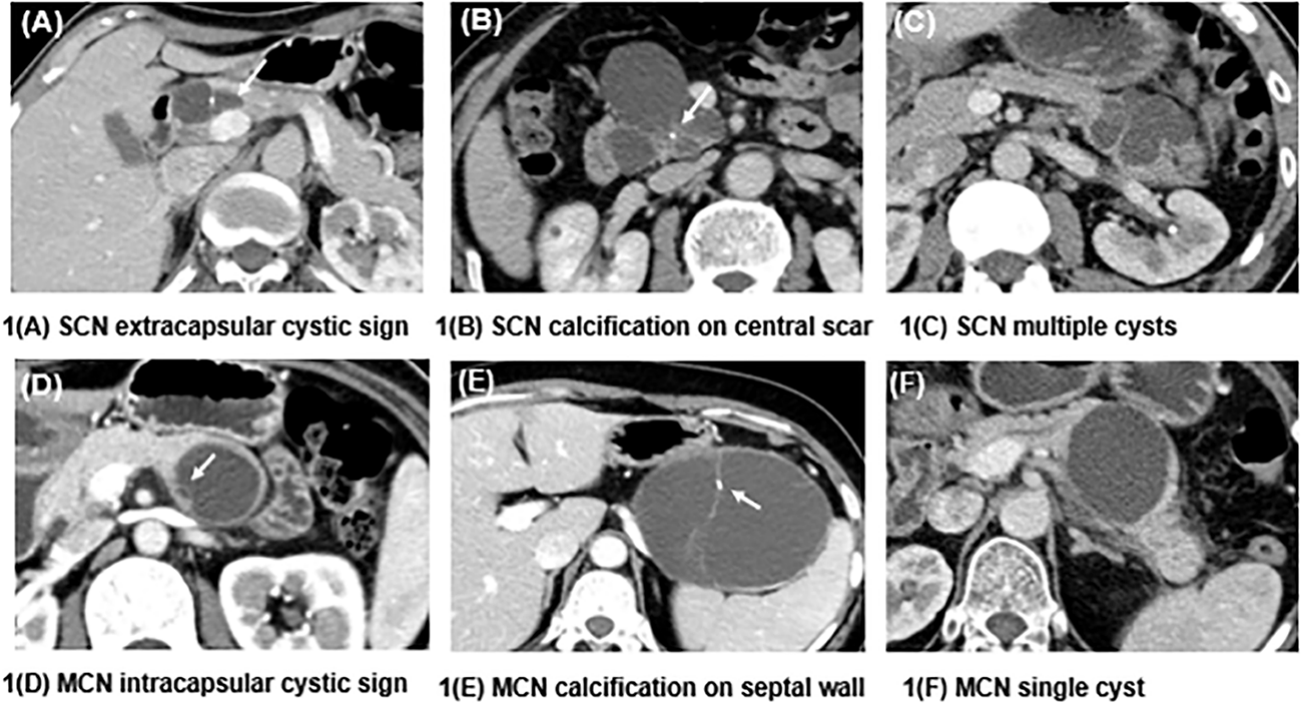
Differences in the characteristics of SCNs and MCNs. **(A)** shows a macrocystic SCN in the head of the pancreas, and a small cyst can be seen outside the mother cyst (white arrow), called the extracapsular cystic sign. **(B)** shows an SCN presenting as a central scar with dotted calcification, which is typical of this kind of neoplasm. **(C)** presents an SCN with multiple cysts that is difficult to diagnose. **(D)** shows a septal wall inside an MCN, which forms a small cyst called the intracapsular cystic sign. **(E)** depicts an MCN with calcification on the septal wall. **(F)** shows an MCN with a single cyst and a smooth contour.

The imaging features were evaluated by two radiologists (3 and 5 years of experience in pancreatic imaging) who were unaware of the pathology of the lesions. Any arguments were settled by consulting with a third radiologist with 31 years of experience in pancreatic imaging. Demographic data, such as sex, age, symptoms, and tumor markers, were collected. The following radiologic features were included: maximum diameter, location, central scar, calcification (on the cyst wall or septum vs on the central scar or noncyst wall), the presence of small cysts (extracapsular cystic sign: a small cyst outside the main cyst; intracapsular cystic sign: a small cyst inside the main cyst), cystic wall thickening (≥3 mm), and tumor morphology (single cyst or multiple cysts) (**Figure 1**).

### Feature Extraction

CT texture features were extracted from the portal venous phase using MaZda software (version 4.6, www.eletel.p.lodz.pl/programy/mazda) (25). Before extraction, all images were processed with standardized grayscale levels to reduce the impact of changes in imaging contrast and brightness. ROIs were drawn in the maximum diameter of the lesion by consensus of the two evaluating radiologists. The intraclass correlation coefficient (ICC) was calculated to assess the stability and reproducibility of the extracted features. Ten patients (5 SCNs and 5 MCNs) were selected randomly, and the ROI was drawn again two months later by two radiologists. An ICC value of at least 0.9 was considered as stable (26).

### Feature Selection

**Figure 2:**
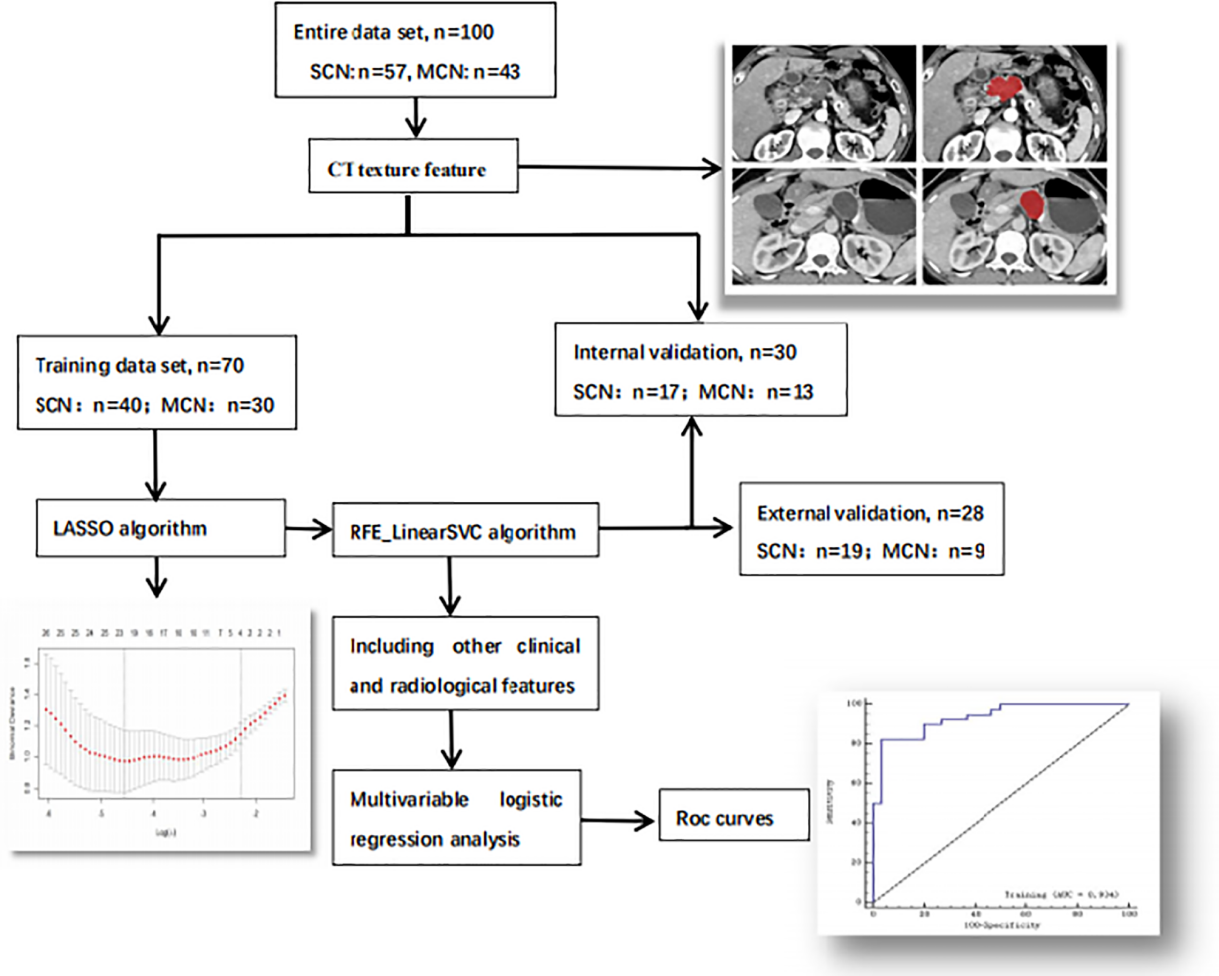
Workflow of the research. The workflow can be divided into four parts: image acquisition, texture feature extraction, texture feature selection and model construction.

The group of 100 patients from center 1 was randomly divided into a training cohort and an internal validation cohort at a 7:3 ratio, while patients from center 2 were used to construct an external validation cohort (n = 28). The workflow is illustrated in **Figure 2**.

A two-step method was performed for texture features selection.

The training dataset was used to select the texture features. The least absolute shrinkage and selection operator (LASSO) algorithm minimizes the residual sum of squares, sets a bound on the sum of the absolute values of the coefficients, and can be used for reducing the dimensions of high-dimensional data (27). Thus, LASSO was implemented by 10-fold cross-validation for feature reduction, and the minimum λ value was calculated to determine the number of selected features. According to the weighted logistic regression coefficient corresponding to each selected feature, the linear mathematical formula contained the score of the radiomics label for each patient was obtained. The formula is listed as follows:


$$Radiomics\_score=w_{0}+w_{1}x_{1}+\text{...}w_{n}x_{n}$$

where *w_n_* denotes to the respective coefficients and *x_n_* denotes the selected features.

The selected features were analyzed by Student’s t-test or the Mann-Whitney U test to eliminate features without significant differences, and then a recursive feature-elimination linear support vector machine (RFE_LinearSVC) was used for further feature selection. RFE_LinearSVC is a powerful method for identifying predictive factors accurately and has consistently outperforms other algorithms in feature selection (28). The internal and external validation cohorts were adopted to verify the performance of the selected features in distinguishing SCNs from MCNs.

### Statistical Analysis

Continuous variables were presented as the median (25-75%), and differences between them were assessed using the Mann-Whitney U test. Categorical variables were expressed as frequencies (%), using Chi test. Multivariable logistic regression with 5-fold cross-validation was conducted for training both the mixed model (radiological features combined with extracted texture features) and the radiological model to identify independent factors and establish diagnostic models. The corresponding receiver operating characteristic (ROC) curves were evaluated, and the prediction efficiency of the two models was quantified by the area under the curve (AUC), the 95% confidence interval (95% CI), sensitivity and specificity.

Clinical and imaging data were analyzed using SPSS 23.0 software, and p < 0.05 was considered as statistically significant. R 3.6.1 software with “glmnet”, “Matrix”, “foreach” and”ggplot2” packages was used to implement the LASSO algorithm. The YITU AI Enabler was applied to implement the RFE_LinearSVC algorithm, and build models were built using python pyradiomics (version 3.0.1) and the scikit-learn (version 0.22) package.

## Results

### General Clinical Information and Imaging Features Among SCNs and MCNs

The demographic data of the participants and their imaging features are summarized in **Table 1**. SCNs were more frequently observed among older women than MCNs (median age of 54 vs 47 years, p < 0.05). The vast majority of MCNs were found in women (90.7%) and were located in the body/tail (86%), while SCNs could occur anywhere in the pancreas equally (p < 0.05). The diameter of the SCNs was generally smaller than that of the MCNs (38.3 mm vs 53.1 mm, p = 0.009), and SCNs were often characterized by characteristic central scars, which could have calcifications, while the calcifications of MCNs often occurred on the cyst wall or septum (p < 0.05). The most characteristic manifestation of MCNs was the intracapsular cystic sign, while the extracapsular cystic sign was more frequent among SCNs. MCNs were more prone to thickening of the cyst wall (14% vs 1.8%, p=0.024) than SCNs. Finally, there was no significant difference between SCNs and MCNs in terms of symptoms, tumor markers, or tumor morphology (p > 0.05).

**Table 1:** Comparison of the clinical information and imaging features between SCNs and MCNs.

Variables	SCNs (n=57)	MCNs (n=43)	P value
Age (years)	54 (44.3-61.3)	47 (33-54)	0.009^*^
**Gender**			0.042^**^
Male	14 (24.6)	4 (9.3)	
Female	43 (75.4)	39 (90.7)	
Symptomatic	11 (19.3)	5 (11.6)	0.300
Tumor maker	4 (7.0)	4 (9.3)	0.476
**Location**			<0.001^**^
Head/neck	26 (45.6)	6 (14.0)	
Body/tail	31 (54.4)	37 (86.0)	
Largest diameter (mm)	38.3 (23.9-52.7)	53.1 (32.2-69.5)	0.009^*^
Central scar	16 (28.1)	0 (0)	<0.001^**^
**Calcification**			<0.001^**^
None	42 (73.7)	34 (79.1)	
On cyst wall	0 (0)	8 (18.6)	
On non-cyst wall	15 (26.3)	1 (2.3)	
**Combined with small cyst**			0.003^**^
None	53 (93.0)	32 (74.4)	
Intracapsular cystic sign	0 (0)	8 (18.6)	
Extracapsular cystic sign	4 (7.0)	3 (7.0)	
**Cystic wall thickening**			0.024^**^
<3mm	56 (98.2)	37 (86.0)	
≥3mm	1 (1.8)	6 (14.0)	
**Tumor morphology**			0.194
Single cyst	12 (21.1)	14 (32.6)	
Multiple cysts	45 (78.9)	29 (67.4)	

^*^means P value has significance by using Mann-Whitney U test; ^**^means P value has significance by using Chi test.

### Feature Extraction and Selection

A total of 271 texture features were extracted: 9 histogram features, 220 gray-level cooccurrence matrix (GLCM) features, 5 gradient features, 5 autoregressive model-based features, 20 run-length matrix features and 12 wavelet features. The ICC value was 0.952 for all features, which was considered stable.

**Figure 3:**
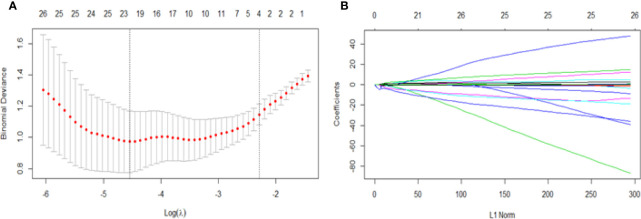
Feature selection for the LASSO algorithm. **(A)** The figure shows binomial deviance (y-axis) plotted against log (λ) (x-axis). The left dotted vertical line is drawn at the optimal value of λ (min λ value = 0.0106, log (λ) = -4.5468), where the model provides the best fit of the data, corresponding to the number of selected features (23). The right vertical dotted line represents the value of λ that yields the best minimum deviation value (minimum λ standard deviation value = 0.1011, log (λ) = - 2.2912). **(B)** LASSO coefficient profiles for all features, which shows that the coefficients of 271 texture features changes with the final selections of different numbers of features.

**Figure 4:**
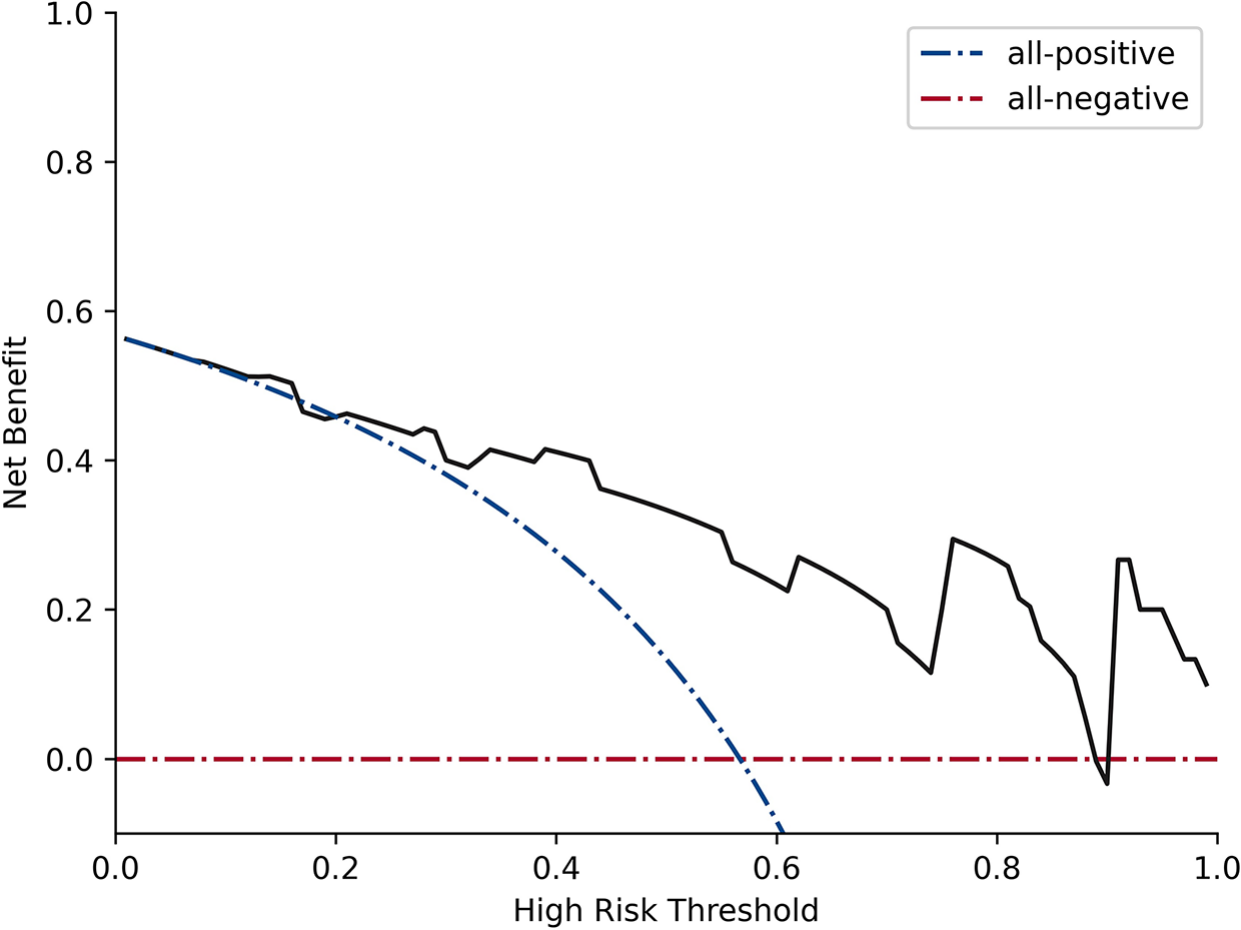
Decision curve analysis for RFE_LinearSVC. The black line represents the true divisional capacity in distinguishing SCNs from MCNs.

**Figure 5:**
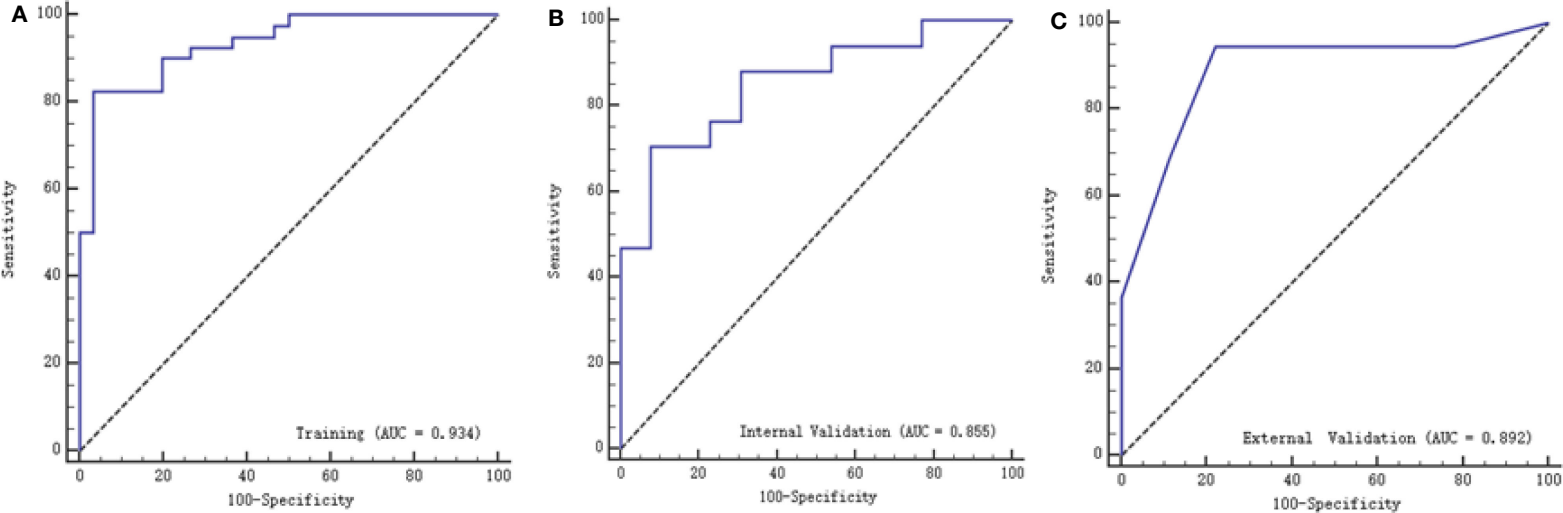
ROC curves of texture features with the training, internal validation and external validation cohorts. **(A)** represents the training group with AUC of 0.934, **(B)** represents the internal validation group with AUC of 0.855, while **(C)** represents with external validation group with AUC of 0.892.

Twenty-three texture features were extracted by the minimum λ value of 0.0106, which was determined through 10-fold cross-validation method using the LASSO algorithm (**Figure 3**). The linear formula contained the score of the radiomics label for each patient was listed in **Supplementary Material 1**. After removing 10 irrelevant statistical variables, 13 texture features were eventually included in the RFE_LinearSVC algorithm as follows: Skewness, S(2,2)DifVarnc, S(2,-2)InvDfMom, S(5,5)InvDfMom, S(4,4)SumEntrp, S(5,5)SumAverg, S(5,5)SumVarnc, Vertl_GLevNonU, 45dgr_GLevNonU, GrNonZeros, Teta1, Teta2, and Teta4. The heat map is shown in **Supplementary Material Figure 1**. Finally, 5 features (Skewness, GrNonZeros, S(2,-2)InvDfMom, S(5,5)InvDfMom, and S(5,5)SumVarnc) were selected with RFE_LinearSVC, and the corresponding decision curve is depicted in **Figure 4**. For the training group, the AUC was 0.934 (95% CI: 0.848-0.980), sensitivity was 90%, and specificity was 76.7%. For the internal and external validation cohorts, the AUCs were 0.855 (95% CI: 0.679-0.956) and 0.892 (95% CI: 0.716-0.977), the sensitivity was 82.4% and 94.7%, and the specificity was 69.2% and 77.8%, respectively (**Figure 5**).

### Multivariable Logistic Regression Models

**Figure 6:**
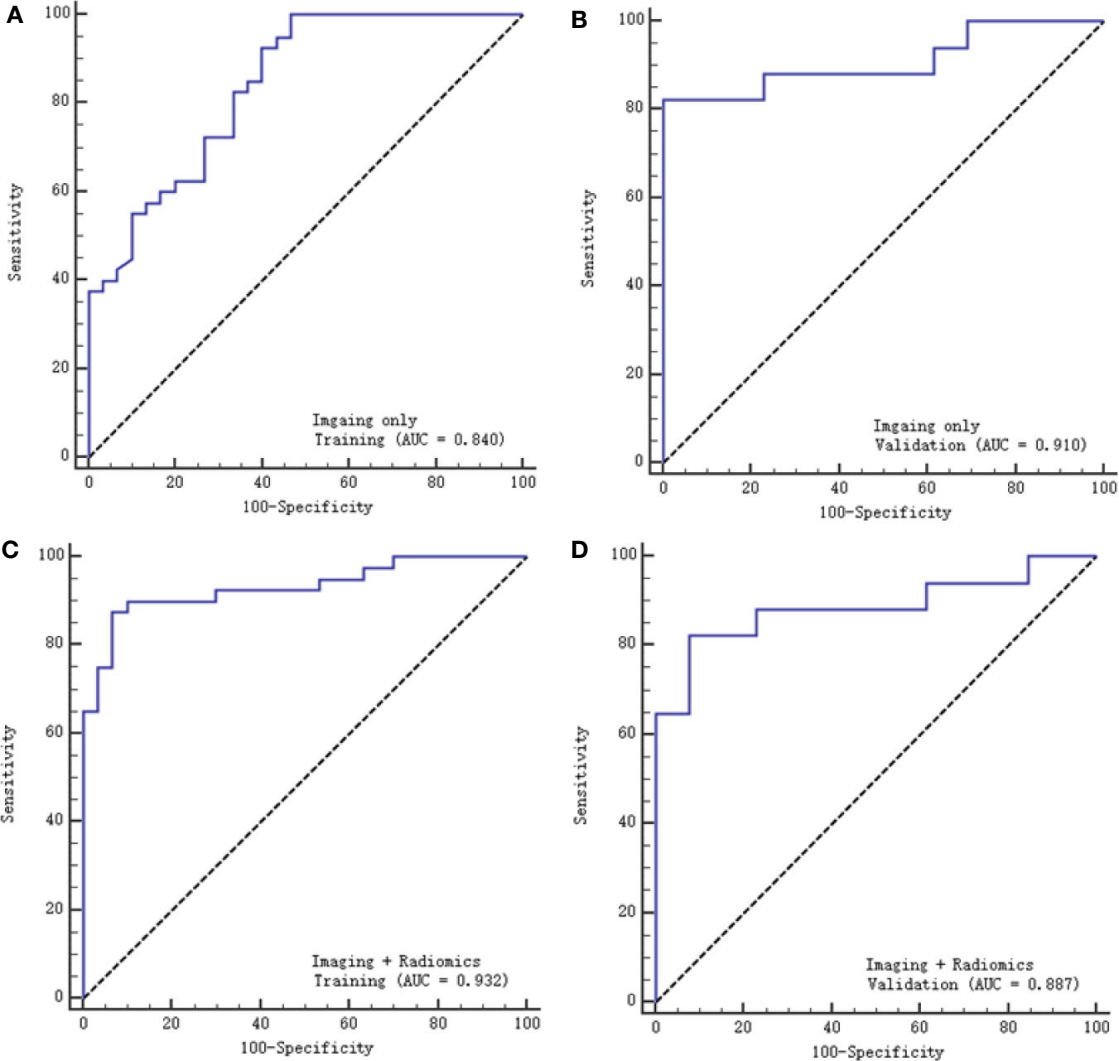
ROC curves of the two models with the training and validation cohorts. **(A, B)** represent the imaging model alone, respectively, and **(C, D)** represent the imaging and radiomics models.

A multivariable logistic regression model combining radiological features and selected texture features was established to differentiate SCNs from MCNs. The AUC was 0.932 (95% CI: 0.845-0.978) and 0.887 (95% CI: 0.0.718-0.973), the sensitivity was 87.5% and 90%, and the specificity was 82.4% and 84.6% with the training and validation cohorts, respectively. Another model based on radiological features alone was also constructed. The AUC was 0.84 (95% CI: 0.732-0.916) and 0.91 (95% CI: 0.747-0.983), the sensitivity was 75% and 66.7%, and the specificity was 82.4% and 77% with the training and validation cohorts, respectively (**Figure 6**).

## Discussion

Radiomics appeared to be superior to conventional clinical and radiologic approaches in differentiating the type of PCLs, while the combination of radiomic features and clinical or imaging features may possibly optimize the predictive accuracy of the model (24). Our most compelling result was that we successfully built multiple logistic regression models to differentiate SCNs from MCNs. The logistic model combining radiological features and enhanced CT texture features had an excellent performance, with an AUC of 0.932, compared with the model built with imaging features only, with an AUC of 0.84.

Imaging performance is the most intuitive approach for distinguishing SCNs from MCNs. SCNs can appear in any part of the pancreas, while the vast majority of MCNs (>90%) occur in the body or tail of the pancreas. Since SCNs have always been misdiagnosed as MCNs or other malignant lesions, several special radiologic features have been summarized to help make an accurate diagnosis, such as the typical honeycomb sign (4), petal sign (29), lobulation sign (30) and extracapsular cystic sign (31). External lobulations appear more frequently in SCNs than in MCNs, and the extracapsular cystic sign also suggests an SCN, while the intracapsular cystic sign is a typical presentation of MCNs, which corresponds to other studies (4, 30, 31).

Although radiological manifestations enable an accurate diagnosis in characteristic cases or provide higher priority to less typical cases, SCNs, especially oligocystic types, are difficult to differentiate from MCNs (7, 32). MCNs present as mildly septate, large cystic neoplasms with smooth unilocular contours, and their cystic wall can be thick and enhanced and accompanied by dotted or arcuate calcifications (33). Surgery should be performed when certain MCN diagnoses are made because these lesions have malignant potential (10). Our study successfully established a diagnostic model based on imaging features. After training the model through logistic regression, the resulting AUC values were 0.84 and 0.91 with the training and validation groups, respectively, suggesting good discriminability when distinguishing SCNs from MCNs. Lee et al. (32) described the MRI features of SCNs and MCNs and found that oligocystic SCNs tended to be smaller lesions with a lobulated (85.7%) contour and multiple clustered cystic configurations. Manfredi R et al. (7) included 57 patients with SCNs and 26 patients with MCNs in the body-tail of the pancreas, and they described similar MR imaging features as previous studies. After analysis, they found that a microcystic appearance, central scarring and lack of peripheral wall enhancement were suggestive of SCNs, whereas a macrocystic appearance, enhancement of the peripheral wall and mural nodules were suggestive for MCNs. However, to overcome diagnostic radiological limitations, radiomics has been recently suggested to differentiate SCNs from MCNs. The combination of radiomics and imaging data may lead to a more precise diagnosis, as described in our study.

Texture analysis has been extensively used for pancreatic tumors as well as PCLs (15–18, 20–22). Yun et al. (17) used CT texture analysis to predict the prognosis of pancreatic cancers and found that texture parameters extracted from preoperative CT images could be used as an independent predictive tool. The grade of pancreatic neuroendocrine tumors could also be predicted accurately; for example, higher skewness and lower kurtosis were identified as risk factors for a higher tumor grade (34). Texture analysis could also identify high-risk disease in patients with IPMNs (35, 36). Xie et al. (22) developed a radiomic model to distinguish macrocystic SCNs (n = 26) from MCNs (n = 31), and their combined model showed better calibration than a single model, as our study showed; however, their sample size was too small and lacked a validation group. Yang et al. (19) included 53 SCNs and 25 MCNs, without external validation, and extracted radiomics features only. After implementing random forest and LASSO methods, they built a diagnostic prediction model to distinguish SCNs from MCNs and obtained AUCs of 0.73 and 0.70 with the training group and the validation group, respectively. Our study also developed a model based on texture features, which achieved an AUC of 0.934 with the training group and 0.855 and 0.892 with the internal and external validation groups, respectively. Textural features derived from enhanced CT images are useful in differentiating SCNs from MCNs and could provide a noninvasive method to identify whether or not surgery is needed. However, the combination of imaging characteristics and texture features outperformed morphological features or texture features alone in Yang’s study (15), which achieved an AUC of 0.893 for the mixed model. They used the LASSO algorithm to select 15 features, and no further algorithm was applied, as in our study. For further feature selection, we used the RFE_LinearSVC method, a machine learning method with an excellent classification performance that leads to superior discrimination (28). The external validation cohort also demonstrated the remarkable capability of the model to differentiate SCNs from MCNs. A radiomics-based method could differentiate SCNs from other PCLs, with an AUC of 0.767, sensitivity of 0.686 and specificity of 0.709 in Wei’s study (23). Shen et al. (20) performed similar work to differentiate SCNs, MCNs and IPMNs, and their random forest classifier achieved the highest accuracy of 84.35 and 79.59% in both the training and validation cohorts. Another study designed an automatic classification algorithm using random forest and conventional neural network ensemble to classify the most common types of PCLs, with an overall accuracy of 83.6% (21).

Several limitations should be emphasized in this study. First, this was a retrospective study with unavoidably inherent selection bias. Second, we only considered the maximal size of the lesion, which may not represent the whole lesion due to tumor heterogeneity. Finally, although the sample size was the largest among similar studies, even larger numbers of patients are required, especially for internal and external validation cohorts, in future investigations.

In conclusion, this study showed that a logistic model combining radiological features and CT texture features is more effective in distinguishing SCNs from MCNs of the pancreas than models built from radiological features alone.

## Data Availability Statement

The original data for this article are not publicly available because of patients information privacy. Requests to access the data should be directed to corresponding author R-SY (risheng-yu@zju.edu.cn).

## Author Contributions

H-YC completed the initial manuscript and designed the whole study; YP and J-YC collected patients and recorded the needed information; X-YD and H-YC collected CT texture features; Y-YL helped collected cases and reviewed the manuscript; W-JC and CL performed the statistics and draw the figure of the manuscript; HY and YZ provided result interpretation; Y-BY performed the control of image quality; G-LS and R-SY revised the manuscript and guaranteed the entire study; All authors did literature research. All authors contributed to the article and approved the submitted version.

## Funding

This study was supported by funding from the National Natural Science Foundation of China (82072032), Major Medical and Health Science and Technology Projects in Zhejiang Province (WKJ-ZJ-2002), Key R&D Projects in Zhejiang Province (2019C03058), Medical Science and Technology Project of the Health Department of Zhejiang Province of China (2019328554, 2021KY091).

## Conflict of Interest

Author CL was employed by Hangzhou YITU Healthcare Technology Co. Ltd.

The remaining authors declare that the research was conducted in the absence of any commercial or financial relationships that could be construed as a potential conflict of interest.

## Publisher’s Note

All claims expressed in this article are solely those of the authors and do not necessarily represent those of their affiliated organizations, or those of the publisher, the editors and the reviewers. Any product that may be evaluated in this article, or claim that may be made by its manufacturer, is not guaranteed or endorsed by the publisher.

## Abbreviations

SCNs, Serous cystadenomas; MCNs, Mucinous cystadenomas; IPMNs, intraductal papillary mucinous neoplasms; PCLs, Pancreatic cystic lesions; LASSO, Least absolute shrinkage and selection operator; RFE_LinearSVC, Recursive feature elimination linear support vector machine; ROC, Receiver operating characteristic; AUC, Area under the curve; CI, Confidence interval; ROI, Region of interest; ICC, Intraclass correlation coefficient; SD, Standard deviation.
