# Supplementary figures and images for: Pancreatic Serous Cystic Neoplasms and Mucinous Cystic Neoplasms: Differential Diagnosis by Combining Imaging Features and Enhanced CT Texture Analysis
Source: Front Oncol. 2021 Dec 23;11:745001. doi: 10.3389/fonc.2021.745001 (PMC8733460; doi:10.3389/fonc.2021.745001)

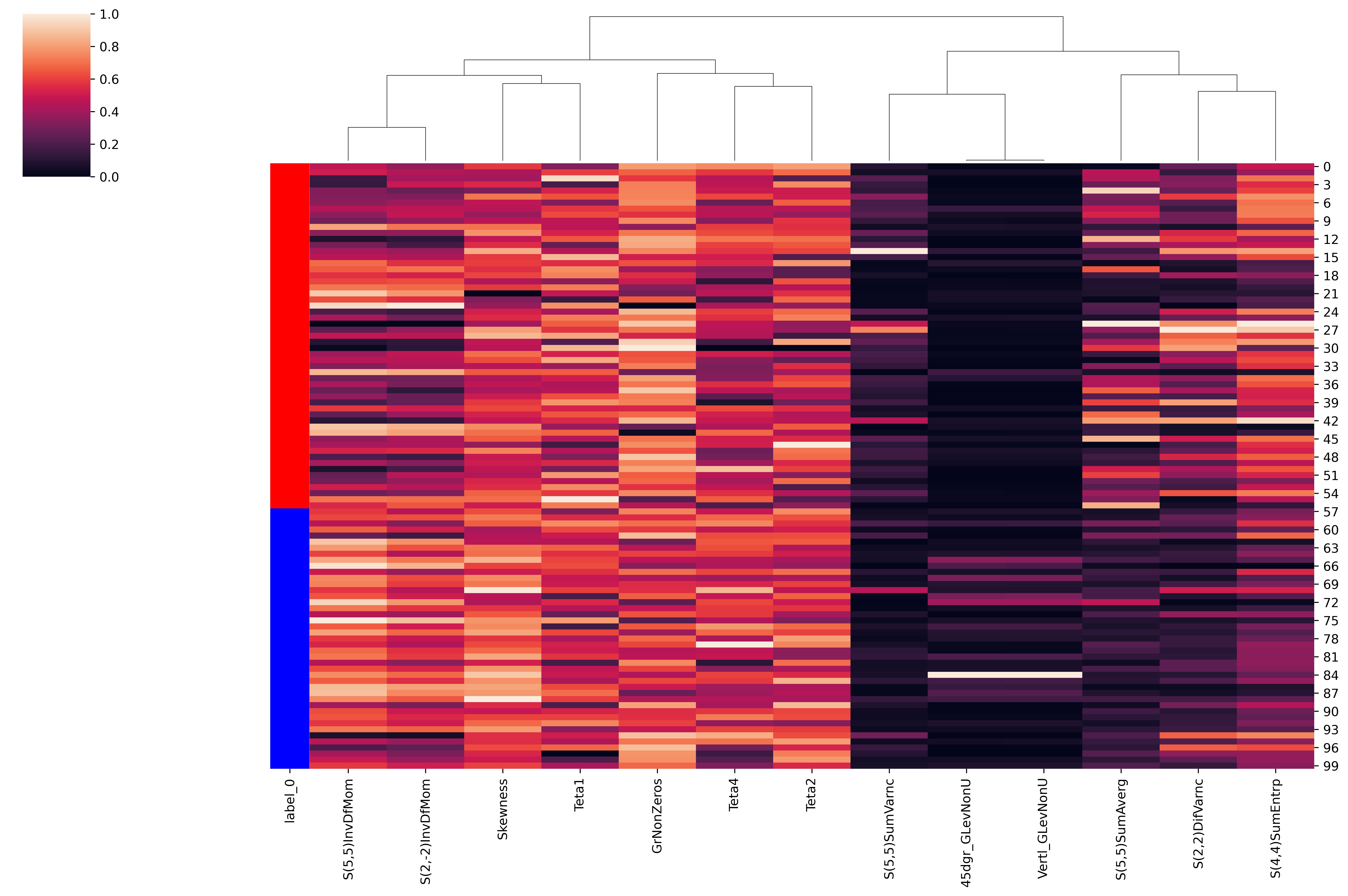

Supplement: Supplementary file 1 [file Image_1.jpeg]
