# Supplementary material for: Pancreatic Serous Cystic Neoplasms and Mucinous Cystic Neoplasms: Differential Diagnosis by Combining Imaging Features and Enhanced CT Texture Analysis
Source: Front Oncol. 2021 Dec 23;11:745001. doi: 10.3389/fonc.2021.745001 (PMC8733460; doi:10.3389/fonc.2021.745001)
Supplement: Supplementary file 2 [file Table_1.doc]

*The score of the radiomics =*

*5.9512+1.466*Skewness+2.764*S(1,1)Correlat+4.2*S(1,-1) DifEntrp+0.834*S(2,2)Correlat-0.385*S(2,2)DifVarnc-13.092*S(2,-2)InvDfMom-6.7*S(4,4)SumEntrp+9.196*S(5,5)InvDfMom-0.007*S(5,5)SumAverg-0.002*S(5,5)SumVarnc+0.002*Vertl_GLevNonU+0.001*45dgr_GLevNonU-0.017*GrKurtosis+4.931*GrNonZeros-12.428*Teta1-7.618*Teta2+2.009*Teta4+0.0003*WavEnLH_s-1+0.054*WavEnHH_s-1+0.002*WavEnLH_s-2+0.104*WavEnHH_s-2-0.004*WavEnLH_s-3+0.06*WavEnHH_s-3*
